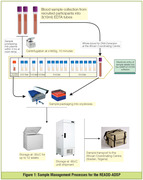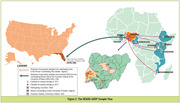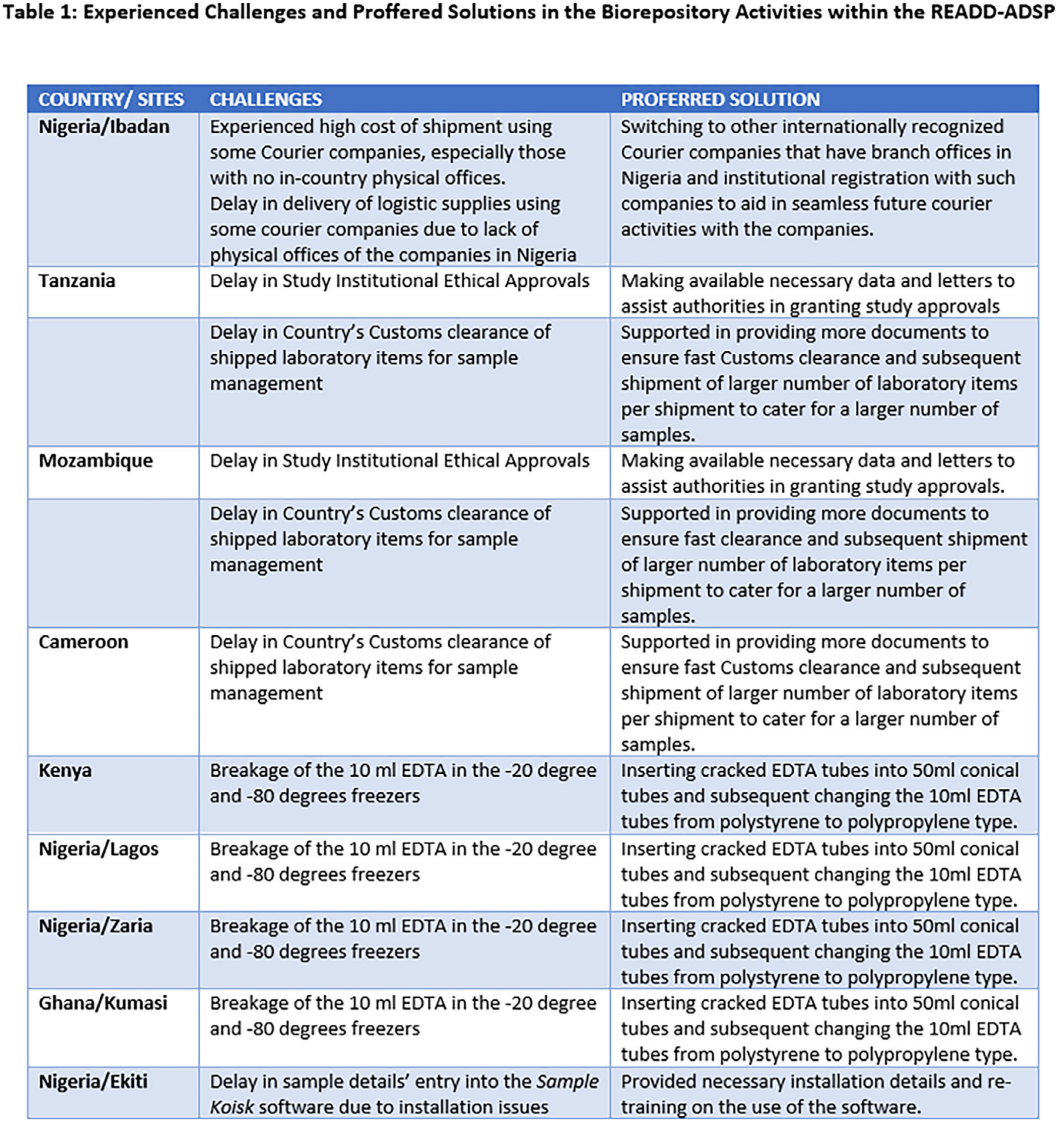# Biobanking and Collaborative Multicenter AD Research in Africa: Preliminary Experience from the Recruitment and Retention for Alzheimer’s Disease Diversity Genetic Cohorts in the Alzheimer’s Disease Sequencing Project

**DOI:** 10.1002/alz.086610

**Published:** 2025-01-09

**Authors:** Kazeem S. Akinwande, Patrice L. Whitehead, Samuel Diala, Larry D. Adams, Motunrayo Coker, Inna Logvinsky, Jovita D. Inciute, Brian W. Kunkle, Osi Adeleye, Mayowa Ogunronbi, Anthony J. Griswold, Jacob L McCauley, Reginald Obiako, David Ndetei, Paul Olowoyo, Albert Kwaku Akpalu, Fred Stephen Sarfo, Biniyam A. Ayele, Albertino Damasceno, Kamada Lwere, Judith Boshe, Kolawole Wahab, Alfred Kongnyu NJAMNSHI, Olayemi Balogun, Pascalyne Nyamai, Tunde Ajibola, William Kudzi, Richard Adjei‐Boateng, Tsehayneh Kelemu, Celia Novela, Rogers Kamulegeya, Happiness Kumburu, Abduraheem Jimoh, Zafack A. Dacein, Roosevelt Anyanwu, Njideka U Okubadejo, Mayowa O Owolabi, Raj Kalaria, Adesola Ogunniyi, Jeffery M. Vance, Rufus O. Akinyemi, Margaret A. Pericak‐Vance

**Affiliations:** ^1^ Institute for Advanced Medical Research and Training, College of Medicine, University of Ibadan, Ibadan, Oyo State Nigeria; ^2^ Federal Medical Centre, Abeokuta, Ogun State Nigeria; ^3^ John P. Hussman Institute for Human Genomics, University of Miami Miller School of Medicine, Miami, FL, USA, Miami, FL USA; ^4^ John P. Hussman Institute for Human Genomics, University of Miami Miller School of Medicine, Miami, FL USA; ^5^ University of Miami, Miller School of Medicine, Miami, FL USA; ^6^ University of Miami Miller School of Medicine, Miami, FL USA; ^7^ University of Miami, Miami, FL USA; ^8^ Ahmadu Bello University, Zaria, Kaduna Nigeria; ^9^ Africa Mental Health Research and Training Foundation, Nairobi Kenya; ^10^ Ekiti State University Teaching Hospital, Ekiti, Ekiti Nigeria; ^11^ University of Ghana, Accra Ghana; ^12^ Komfo Anokye Teaching Hospital, Kumasi Ghana; ^13^ Addis Ababa University, Addis Ababa, Addis Ababa Ethiopia; ^14^ Eduardo Mondlane University, Maputo Mozambique; ^15^ Makerere University, Kampala Uganda; ^16^ Kilimanjaro Christian Medical Center, Moshi Tanzania, United Republic of; ^17^ Univeristy of Ilorin Teaching Hospital, Ilorin, Kwara Nigeria; ^18^ Brain Research Africa Initiative, Yaounde, Centre Cameroon; ^19^ Ekiti State University Teaching Hospital, Ekiti Nigeria; ^20^ Addis Ababa University, Addis Ababa Ethiopia; ^21^ University of Yaounde, Yaounde Cameroon; ^22^ College of Medicine, University of Lagos, Lagos Nigeria; ^23^ African Dementia Consortium, Lagos Nigeria; ^24^ College of Medicine, University of Ibadan, Ibadan, Oyo State Nigeria; ^25^ Newcastle University, Newcastle upon Tyne United Kingdom; ^26^ Neuroscience and Aging Research Unit, Institute of Advanced Medical Research and Training, College of Medicine, University of Ibadan, Ibadan Nigeria

## Abstract

**Background:**

The burden of Alzheimer’s disease and related dementias is growing fast in Africa. The Recruitment and Retention for Alzheimer’s Disease Diversity Genetic Cohorts in the Alzheimer’s Disease Sequencing Project (READD‐ADSP) has commenced recruitment of 5000 African participants (AD and cognitively unimpaired individuals) to generate genomic and biomarker data to better characterize AD genetic architecture in Africa. Participating countries, part of the African Dementia Consortium (AfDC) include Nigeria, Ghana, Benin, Cameroon, Uganda, Kenya, Ethiopia, Tanzania, and Mozambique. This abstract focuses on the biobanking activities, including the challenges and lessons learnt in LMIC locales.

**Method:**

A Standard Operating Procedure (SOP) was developed for all biobanking activities, supplemented with monthly Zoom meetings to discuss efforts across all sites. Blood samples were collected and transported to the African Coordinating Centre (ACC: Ibadan, Nigeria) where DNA extraction, plasma isolation, and long‐term biospecimen storage occurred (Figure 1). Small aliquots of DNA and plasma were sent to the Hussman Institute at the University of Miami (HIHG‐UM) (Figure 2) for whole genome sequencing and biomarker analysis. The biobanking activities were guided by the International Society for Biological and Environmental Repositories (ISBER) benchmarks for internationally accepted best practices in biorepository science.

**Result:**

Sample collection kits and extraction materials were centrally procured at UM, shipped to the ACC, then distributed. Standardized blood sample collection and processing (e.g., use of sample kiosk software, short‐term (<12 weeks) biospecimen storage at ‐20^o^C and long‐term storage at ‐80^o^C, and shipment from African sites to ACC) were achieved. DNA was extracted at ACC and shipped together with plasma to HIHG‐UM. The duration of intercontinental shipment (USA to‐and‐from Nigeria) was 18.5+/‐2 days while shipment within Africa was 5+/‐3.5 days. Challenges encountered included delays with institutional ethics approval, custom clearance at borders, high shipping costs between African countries, and occasional breakage of stored blood tubes. Innovative solutions were devised to mitigate these challenges (Table 1). Initial shipment of samples to the HIHG‐UM included >200 DNA samples of excellent quality.

**Conclusion:**

Our biobanking experience in a LMIC setting demonstrates the feasibility of establishing a successful African biobanking network, an important infrastructure to support AD/ADRD research in Africa.